# Between a Rock and a Hard Place: An Epigenetic-Centric View of Testicular Germ Cell Tumors

**DOI:** 10.3390/cancers13071506

**Published:** 2021-03-25

**Authors:** Ratnakar Singh, Zeeshan Fazal, Sarah J. Freemantle, Michael J. Spinella

**Affiliations:** Department of Comparative Biosciences and the Carle Illinois College of Medicine and the Cancer Center of Illinois, University of Illinois at Urbana-Champaign, Urbana, IL 61801, USA; rsingh02@illinois.edu (R.S.); fazal2@illinois.edu (Z.F.); sarahf@illinois.edu (S.J.F.)

**Keywords:** testicular cancer, epigenetics, embryonal carcinoma, cisplatin, resistance, testicular germ cell tumors, DNA methylation

## Abstract

**Simple Summary:**

This minireview focuses on the role of epigenetics in testicular cancer. A working model is developed that postulates that epigenetic features that drive testicular cancer malignancy also enable these tumors to be cured at a high rate with chemotherapy. Chemoresistance may occur by epigenetic uncoupling of malignancy and chemosensitivity, a scenario that may be amenable to epigenetic-based therapies.

**Abstract:**

Compared to many common solid tumors, the main genetic drivers of most testicular germ cell tumors (TGCTs) are unknown. Decades of focus on genomic alterations in TGCTs including awareness of a near universal increase in copies of chromosome 12p have failed to uncover exceptional driver genes, especially in genes that can be targeted therapeutically. Thus far, TGCT patients have missed out on the benefits of targeted therapies available to treat most other malignancies. In the past decade there has been a greater appreciation that epigenetics may play an especially prominent role in TGCT etiology, progression, and hypersensitivity to conventional chemotherapy. While genetics undoubtedly plays a role in TGCT biology, this mini-review will focus on the epigenetic “states” or features of testicular cancer, with an emphasis on DNA methylation, histone modifications, and miRNAs associated with TGCT susceptibility, initiation, progression, and response to chemotherapy. In addition, we comment on the current status of epigenetic-based therapy and epigenetic biomarker development for TGCTs. Finally, we suggest a unifying “rock and a hard place” or “differentiate or die” model where the tumorigenicity and curability of TGCTs are both dependent on common but still ill-defined epigenetic states.

## 1. Introduction

Human germ-cell tumors are a heterogeneous group of neoplasms, which occur in the gonads, both the ovaries and the testes, and in distinct extragonadal sites along the midline of the body and brain [1]. Approximately 90–95% of testicular cancers correspond to pathologies originating from the germ cell lineage called testicular germ cell tumors (TGCTs) [2]. In 2016, the World Health Organization (WHO) reported a comprehensive classification system for TGCTs that considers the germ cell involved, the embryonic and extra-embryonic lineages present, the histological composition of the tumor and the developmental potential of the cell of origin [3]. Based on this classification system, TGCTs are divided into two main groups (i) tumors derived from germ cell neoplasia in situ (GCNIS); and (ii) tumors considered not to be derived from GCNIS [3,4]. GCNIS related TGCTs (also known as type II TGCTs) occur in adolescents and young adults and are histologically subdivided into seminoma and nonseminoma (that is, embryonal carcinomas, teratomas, yolk sac tumors and choriocarcinomas) that can be pure or intermixed. Non-GCNIS related TGCTs include mainly pediatric or prepubertal tumors (also known as type I TGCTs) that include benign teratoma and malignant yolk sac tumors, and spermatocytic tumors of the testis (also known as type III TGCTs) that predominantly affect men >50 years old [5].

TGCTs are rare solid tumors which account for 1% of cancers in men, but the most common solid malignancy of young males between the ages of 15 and 44 years [6]. The incidence of TGCTs has been steadily rising in young males [7,8]. Recent statistics estimated that TGCTs have the largest number of new cases among all cancers for males under the age of 34 years in the USA and second largest worldwide [6]. The incidence of TGCTs varies widely with geographic location with the highest incidence in northern European countries and the lowest in African nations [8]. The reasons for these differences are not known but are hypothesized to involve a combination of inherited and environmental factors including exposure to endocrine disruptors in utero [2,9].

In general, TGCT treatment is highly successful. Testicular cancer prognosis is generally favorable, particularly in high-income countries [10]. Mortality rates are higher in low-incident and low-income countries compared to high-incident, high-income countries [11]. Patients diagnosed with a non-metastatic TGCT have a five-year survival of ≥95% [6]. Furthermore, metastatic testicular cancer can be cured with conventional cisplatin-based therapy at a rate approaching 80% while most common solid tumors are fatal in the metastatic setting [12,13] However, 30% of TGCT patients are refractory or develop resistance to cisplatin-based therapy and approximately 50% of these patients die from progressive disease [14,15]. There is a clinical need for new more targeted approaches for TGCTs for the treatment of cisplatin refractory disease and for the avoidance of cisplatin related toxicities which can be significant and lifelong [16,17,18,19]. 

TGCTs are at the crossroads of developmental and neoplastic processes [2,5,20]. TGCTs are characterized by substantial histological heterogeneity, despite a uniform cytogenetic profile including uniform chromosomal 12p amplifications [21]. The etiology of TGCTs is uncertain compared to many somatic cancers [22,23]. In general, the low frequency of somatic mutations in TGCTs, especially in nonseminomas compared to other cancers, suggest that epigenetic mechanisms play an especially prominent role in their pathogenesis [24,25]. TGCTs also possess distinct epigenetic states compared to somatic cancers that reflect their primordial germ cell (PGC) origins that underlie the unique biology of these tumors, including their curability [2,26,27,28]. Distinct epigenetics may render TGCTs distinctly susceptible to epigenetic targeted therapies [29,30,31,32]. 

In this mini-review, we summarize the main epigenetic features of testicular cancer, with a focus on DNA methylation, histone modifications, and miRNAs and their involvement in testicular carcinogenesis and response to chemotherapy. In addition, we address the current status of epigenetic-based therapy and epigenetic biomarker development for TGCTs. Finally, we suggest a unifying model where the tumorigenicity and curability of TGCTs are both dependent on common but still ill-defined epigenetic states.

## 2. Risk Factors and Pathogenesis of TGCTs

**Figure 1:**
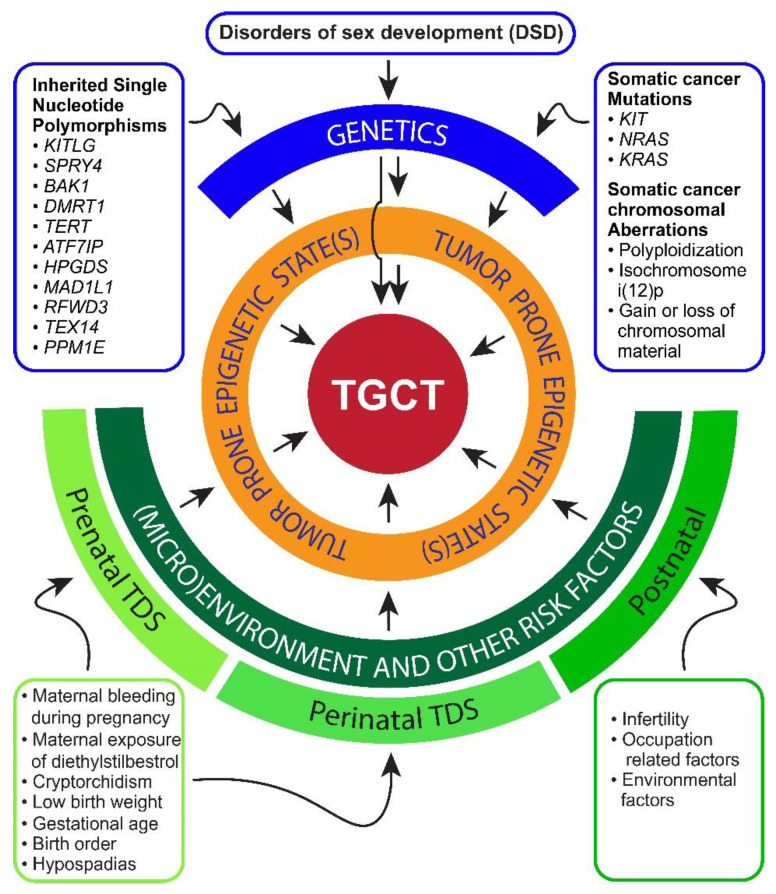
An epigenetic-centric view of testicular germ cell tumors (TGCT) etiology. While genetics undoubtedly play a role in TGCT development, epigenetic altering factors in the microenvironment of the testis from both internal and external sources may be a more important driving factor. The model also suggests that even inherited genetic germline variants and somatic cancer alterations may participate in establishing tumorigenic epigenetic state(s) of TGCTs. DSD, disorders of sex development, TDS, testicular dysgenesis syndrome.

The etiology of TGCTs is still largely unknown [1,2]. Despite a strong familial hereditary component, robust genetic germline risk alleles have not been found for TGCTs [33]. However, several low penetrant, inherited germline alleles have been identified including *KITGL*, *SPRY4*, and *BAK1* suggesting a polygenetic nature to TGCT risk [34,35,36,37,38,39,40,41,42,43,44,45]. Interestingly, some of these risk alleles are also associated with regulating epigenetics or germ cell differentiation. The incidence of TGCTs, once extremely rare, has increased significantly and steadily, especially in industrialized nations suggesting a potential role for environmental toxicants [6,7,8]. For example, the annual number of TGCT cases has doubled worldwide since the 1950s and continues to rise [6,7,8]. Several features predict impact from the microenvironment of male germ cell development, including cryptorchidism, hypospadias, prior testicular cancer, impaired spermatogenesis, a family history of testicular cancer, Down’s syndrome, low birth weight, premature birth, birth order, bleeding during pregnancy, estrogen exposure during pregnancy, high maternal age, and neonatal jaundice that are proposed risk factors for TGCTs (Figure 1) [46,47,48,49,50,51,52,53,54,55,56]. Associations and co-occurrences of two or more factors in the same individual and at the population level has led to the hypothesis that a testicular dysgenesis syndrome (TDS), likely originating in utero, is a key predisposing factor for testicular cancer [57,58]. The increased incidence of TDS is suggested to be associated with exposure to environmental agents such as endocrine disrupters and certain toxic industrial chemicals. Sex steroid hormones (especially androgens) have an important role in urogenital development. Studies have suggested that an in utero hormonal disturbance increases the risk of TGCTs [59,60,61]. A substantial proportion of germline, genetic disorders of sex development (DSD), associated with fetal androgen insufficiency, are also associated with increased risk of germ cell malignancy [62,63]. The recent realization that environmental factors likely play a significant role in the pathogenesis of testicular cancer has led to a “genvironmental” model suggests that an interaction between environmental factors (that mediate epigenetic changes) and genetic alterations can explain the shifting epidemiology of TGCTs and its intricate association with DSD and TDS [64] (Figure 1).

Primordial germ cells (PGCs), precursors of sperm and eggs, generate a new organism that is capable of creating endless new generations. Defects in PGC differentiation lead to germ cell tumors [20,65]. Under normal conditions PGCs further differentiate and form gonocytes [66]. Arrested gonocytes that fail to mature to spermatogonia lead to the tumors of young adults (seminomas and non-seminomas) which are preceded by GCNIS [20,65]. GCNIS cells gradually acquire secondary somatic genomic changes and transform to malignant germ cells while adapting to the post-pubertal testis niche [67]. Due to common origins, GCNIS, seminoma and embryonal carcinoma (EC) have many morphological similarities with PGCs and early gonocytes. Expression of *POU5F1* (OCT4) and *NANOG*, which are known for their association with pluripotency in embryonic stem cells are also characteristic of GCNIS and derived invasive testicular cancers [68]. Other markers, including placental-like alkaline phosphatase (*PLAP*), the stem cell factor receptor *KIT*, and *TFAP2C* (AP2γ) also provide evidence that GCNIS and its derived cancers are similar to PGCs [20,68]. Two characteristic secondary somatic chromosomal aberrations associated with GCNIS and derived TGCTs are polyploidization and amplification of chromosome 12, which often manifests as an isochromosome i(12)p [21,69]. Other secondary chromosomal abnormalities associated with TGCTs include gain of genetic material on chromosomes 1, 2p, 7, 8, 12, 14q, 15q, 17q, 21q, and X and the deletion of genetic material from chromosomes 4, 5, 11q, 13q, and 18q2 [21,69]. Similarly, multiple passaging of embryonic stem cells has been shown to result in acquired alterations similar to those found in TGCTs and EC, including chromosome 12, 17, and X gain [70]. Genome-wide sequencing indicates secondary somatic gain/amplification of 12p in the majority of patients with gain in 12q, 8q, 22q, and deletion/loss of 11q, 18q, 18p, 9p, 4q, 10q, 5q, 16q, and 19q also occurring with much less frequency [24,25,71]. Despite cytogenetic abnormalities the mutational burden of TGCTs are low compared to somatic cancers. 

Somatic mutation in TGCTs are relatively rare and occur mostly in seminoma. Specific somatic mutations or amplifications in tumor DNA have been identified in a few genes, but only *KIT*, *KRAS*, and *NRAS* have been implicated repeatedly in different studies, mostly in seminomas [24,25,71]. Gain-of function somatic mutations in *KIT* have been detected in up to 25% of seminomas but are very rare in non-seminoma germ cell tumors [24,25,71]. Allelic inherited germline variation within *KITLG*, the gene encoding the ligand for KIT receptor is the strongest genetic risk factor for TGCTs [34]. Other rare inherited germline inactivating *PDE11A* variants are most likely associated with TGCTs of young adults [72]. Comparing somatic mutations in GCNIS and TGCTs suggest that genetic mutations are likely involved in progression of TGCTs rather than initiating events [73]. It is worth noting that TGCTs possess a high level of genomic instability, including aneuploidy and recurrent copy number changes that likely participate in tumor progression [24,25,71].

Endocrine disrupting chemicals that impair estrogen and androgen signaling during early development are associated with an increased risk of TGCTs. Some occupations, such as firefighting and aircraft maintenance also increase TGCT risk [74,75]. Testicular physiology is well characterized and known to be hormone sensitive. The impact of hormones on the testis is mainly through the androgen and estrogen receptors. Interestingly, some studies have explored the potential association of TGCTs with inherited single nucleotide polymorphisms of the androgen receptor (AR), the estrogen receptors (ER) and genes involved in either synthesis or degradation of gonadal hormones [76,77,78,79]. It is important to remember that potential epigenetic disruptions may not necessarily target PGCs/gonocytes directly but may alter the epigenetic state of the somatic cells of the testis, Leydig and Sertoli cells which produce growth factors and hormones necessary to maintain the proper micro-environment during spermatogenesis.

Thus, from an epigenetic-centric view, epigenetic perturbations that arrest PGC/gonocyte differentiation leading to tumorigenic prone states are dominant events downstream of the many risk factors outlined in this section including environmental exposures, inheritable genetic factors and developmental reproductive disorders predicted to alter the microenvironment of the developing germ cell (Figure 1). We contend that perhaps the majority of risk factors converge to favor a limited number of cancer driving epigenetic states that in some cases further progress due to somatic genetic mutations including those of *KIT* and *KRAS*. Many decades of focus on somatic genomic alterations in TGCTs including awareness of near universal increase in copies of chromosome 12p have failed to uncover exceptional cancer driver genes. This at least suggests that an epigenetic mediated tumorigenic state or states may be the true driver of TGCTs. 

## 3. Mechanisms of Chemotherapy Sensitivity and Resistance in TGCTs

A defining feature of metastatic TGCTs is a unique hypersensitivity to conventional chemotherapeutics especially to cisplatin-based therapies [12,13]. However, a clinically significant proportion of patients are resistant to cisplatin-based therapy and die from progressive disease [14,15]. This is in part due the lack of targeted therapies for TGCTs which is in turn likely due to a paucity of driver mutations or genomic alterations. Further, while the majority of TGCTs with advanced disease are cured with chemotherapy, this is associated with acute and lifelong toxicities in an exceptionally young patient population [16,17,18,19]. Hence there is a strong rationale to develop alternative and cisplatin-sparing therapies for TGCTs. Similar to etiology and initiation, the mechanisms responsible for TGCT chemosensitivity and resistance have been elusive and appear for the most part to be independent of acquired genetic mutations or alterations [29,80,81,82]. In this section we briefly review traditional mechanisms proposed to account for TGCT chemosensitivity and resistance.

Perhaps the most convincing mechanism for TGCT resistance due to acquired mutation is mutation of p53 or p53 compromised by increased *MDM2* copy number. The p53 gene is notoriously wild-type in most TGCTs unlike the situation for other cancers. This presents a plausible mechanism to account for the hypersensitivity of TGCTs to chemotherapy. While genetic alterations in the p53 pathway do occur in cisplatin resistant cell models and in patients, the percentage is low and does not appear to explain the majority of cisplatin resistant disease [24,71,83,84,85,86]. Other suggested mechanisms of cisplatin resistance include alterations in *PDGFR/PI3K/AKT/PTEN, IGF1R*, Cyclin D1, p21, autophagy and apoptotic pathways [29,80,81,82]. Additional proposed mechanisms include alterations in cisplatin accumulation or increased inactivation of cisplatin by conjugation reactions [29,80,81,82]. The alterations in most of these cases involve expression level changes of pathway components and not fixed acquired mutations or copy number changes.

Another major proposed mediator of cisplatin resistance in TGCTs has been DNA repair and DNA damage response pathways, especially the nuclear excision repair (NER), base excision repair (BER), homologous recombination (HR) and mismatch repair (MMR) pathways [87,88,89,90,91,92,93,94]. This has led to studies suggesting that TGCTs may be vulnerable to PARP inhibitors [95,96]. Again, the argument has been that alterations mostly in the levels of DNA repair and DNA damage response (DDR) pathway components and not common genetic mutations result in either more efficient DNA repair or increased tolerance to DNA damage in cisplatin refractory TGCT cells. Interestingly expression levels of some of these components have been proposed to involve alterations in promoter DNA methylation, thus providing crosstalk between traditional DNA repair/DDR based mechanisms of chemotherapy resistance and epigenetic reprogramming in TGCTs [95,97].

In contrast with traditional/genomic mechanisms of chemoresistance a relatively unexplored explanation involves epigenetics. In an epigenetic-centric view of TGCTs one would postulate that even traditional mechanisms of chemotherapy resistance like dysregulation of the DNA repair and the DNA damage response could in actuality be downstream consequences of altered epigenetic states that provide, for example, more efficient DNA repair or increased tolerance of cellular DNA damage.

## 4. Epigenetic States in Testicular Germ Cell Tumors Associated with Tumorigenicity and Chemosensitivity

Despite involvement of genetic factors, TGCTs are clearly not a disease of accumulating mutations like most solid tumors, and again unlike most cancers, genetic alterations appear to poorly explain the initiation and the unique curability of TGCTs. The paucity of actionable driver mutations in TGCTs suggests that a number of as yet poorly characterized genetic states may mediate key aspects of tumorigenicity and chemotherapy response in TGCTs. In this section we briefly review recent findings related to epigenetic states of TGCTs.

Epigenetic regulation of gene expression takes places at the protein level (post-translational histone modifications), DNA level (DNA methylation), and RNA level (non-coding RNAs). These epigenetic modifications can create a compact chromatin structure (heterochromatin, which is transcriptionally silent) or an open chromatin structure (euchromatin, which is transcriptionally active). These changes in gene expression are known to be influenced by chemical modifications on histone tails or DNA. The most studied epigenetic modifications include DNA methylation, histone acetylation and histone methylation. Dynamic epigenetic changes occur during the male germ cell cycle. PGCs and gonocytes in the male gonad have undergone epigenetic reprogramming which includes erasure and re-establishment of DNA methylation and alterations in histone modifications [98]. Patterns of DNA methylation and histone modifications in TGCTs are distinct from those in somatic cancers and show developmental patterns [20,27]. The third mechanism involved in regulation of gene activity is RNA interference targeting specific mRNA molecules at the posttranscriptional level. Small RNAs, including microRNAs (miRNAs), endogenous small interfering RNAs (endo-siRNAs) and PIWI-interacting RNAs (piRNAs) that may to be involved in the control of male germ cell differentiation may also participate in TGCTs carcinogenesis and chemosensitivity [99,100]. Distinct epigenetic states have also been associated with subtype identity and cisplatin resistance in TGCTs [25,101,102].

### 4.1. Differentiation

While the focus on epigenetics has been relatively recent, the relationship between differentiation, tumorigenicity, and chemosensitivity of TGCTs is well established. Specifically, it is well recognized that the degree of pluripotency or stem-like properties of TGCT histological subtypes roughly correlates with chemosensitivity, with seminoma and EC being the most chemosensitive followed by yolk sac, choriocarcinoma and immature teratoma with intermediate chemosensitivity followed by chemotherapy resistant, mature teratoma [29,80]. Differentiation status of TGCTs is presumed to be driven by epigenetic mechanisms. For example, DNA methylation but not genomic content tracks with differentiation and chemotherapy sensitivity (see below). During chemotherapy of advanced malignant TGCTs often all that remains is benign non-malignant teratoma resistant to cisplatin therapy [103]. Several clinical, animal, and cell line studies have shown a correlation between pluripotency markers OCT4 and NANOG and the degree of TGCT cisplatin sensitivity [104,105,106,107]. Hypoxia and cisplatin treatment of TGCT cells induce OCT4 repression and cisplatin resistance, in one instance correlating with OCT4 regulation of p21 via miR106b [108,109]. Experimentally induced differentiation of (embryonal carcinoma (EC) cells by retinoic acid or depletion of OCT4 also results in cisplatin resistance, further suggesting a tight link between pluripotency and chemosensitivity of TGCTs [104,105,106,107]. Retinoic acid induced differentiation has also been associated with down regulation of antitumor proteins EIR3, NOXA, and PUMA associated with cisplatin resistance [110]. There have also been studies demonstrating epigenetic mediated conversion of seminoma to nonseminoma in a seminoma cell line xenograft [111].

The stem cell of TGCTs, EC, have distinct properties compared to cancer stem cells in other solid tumors. Cancer stem cells in somatic tumors are often resistant to chemotherapy while the pluripotency of EC cells is associated with driving TGCT curability. This distinction is again likely driven in part by the unique epigenetic status of EC and seminoma. While other cancer stem cells have been suggested to possess stem cell properties including the expression of pluripotency markers including OCT4 and NANOG, EC are truly pluripotent and both EC and seminoma express OCT4, NANOG and other pluripotency markers orders of magnitude higher than somatic cancer stem cells [112].

### 4.2. DNA Methylation

DNA methylation is the most studied epigenetic mark in TGCTs and there is relatively strong evidence that DNA methylation is differentially distributed among the histologically distinct subtypes of TGCTs and among cisplatin sensitive and resistant tumors. As described above, TGCTs are believed to arise from PGCs or gonocytes, the stage in germ line development where DNA methylation and parental imprints are erased and totipotency is restored [98]. DNA methylation is an important regulator of gene transcription. Its role in carcinogenesis and chemoresistance has been a topic of considerable recent interest. The methylation of DNA is a major event during germ cell development and differentiation in retrotransposons, repeated regions, and differentially methylated regions (DMRs) of genes.

DNA methylation has been recognized as an important mechanism during TGCT progression and chemosensitivity [113,114,115,116]. TGCT genomic DNA is hypomethylated in comparison to most solid tumors. Like PGCs, GCNIS and seminomas display global DNA hypomethylation [25,101,102]. In contrast, teratomas display CpG methylation levels comparable with somatic tumor entities, while EC possess an intermediate level of CpG methylation and also uniquely possess non-CpG methylation similar to embryonic stem cells [25,101,102]. Methylome studies in TGCTs have shown that GCNIS, seminomas and nonseminomas are consistently demethylated in repetitive LINE1 elements and at imprinted genes and the XIST locus [25,101,102,117]. GCNIS and seminomas are also demethylated at ALU elements while ALU elements are partially methylated in nonseminoma [25,101,102]. Furthermore, locus specific promoter methylation occurs in nonseminoma that is distinct for each histological type, again with a general pattern of increased methylation with increased differentiation status [25,101,102]. This includes evidence that methylation of such genes as *CRIPTO*, *HOXA9 MGMT*, *RASSFIA*, *SCGB3A1*, *CALCA*, *MMP9*, *CSFR1*, and *PTPRC* is associated with distinct TGCT subtypes or poor prognosis [101,114,115,116,118].

There is evidence to suggest that hypermethylation of TGCTs especially EC and other nonseminoma subtypes may be associated with cisplatin resistance. Several specific gene promoters and differentially methylated regions (DMRs) have been associated with inherent and acquired cisplatin resistance in TGCTs and cell lines including *RASSF1A*, *HIC1*, *MGMT*, and *CALCA* [113,114,115,116]. Additionally, cisplatin treatment has been shown to be associated with increased DNA methylation in vivo [114]. Expression levels of DNMTs and TET enzymes responsible for adding and removing DNA methylation have been shown to be dynamically regulated and associated with clinical outcomes in TGCTs [119,120].

Utilizing a series of isogenic cisplatin-resistant cell models we demonstrated a strong association between cisplatin resistance in TGCT cells and a net increase in global CpG and non-CpG DNA methylation spanning regulatory, intergenic, genic, and repeat elements [113]. Integrative transcriptome and methylome analyses revealed a strong negative correlation between gene promoter and CpG island methylation and gene expression in resistant cells. A bidirectional shift between gene promoter and gene body DNA methylation occurred within multiple genes that was associated with upregulation of polycomb targets and downregulation of tumor suppressor genes [113]. These findings suggest that global remodeling of DNA methylation is a key factor in mediating cisplatin hypersensitivity and that chemoresistance of TGCTs is mediated by a complex interplay between DNA methylation and the polycomb pathway (see below) [113].

In the epigenetic centric view, the unique epigenetic states of TGCTs may not only be responsible for tumorigenicity and chemosensitivity but may also be exploitable as therapeutic targets. In this regard DNA methyltransferase inhibitors have been furthest advanced. We and others have shown that EC cells are hypersensitive to the DNA methyltransferase inhibitors decitabine, azacytidine, guadecitabine, and MLo1302 in vitro and in vivo compared to somatic tumors [121,122,123,124,125,126]. This hypersensitivity extends to cisplatin refractory EC and is dependent on high levels of the DNA methyltransferase, DNMT3B [121,122,123,124,125]. Importantly, pretreatment of cisplatin refractory cells in vitro and in vivo could resensitize to cisplatin and this activity was associated with activation of p53 targets and a stress response, repression of pluripotency genes, and activation of genes repressed by DNA methylation [121,122,123,124,125]. A seminoma cell line was also sensitized to cisplatin after azacytidine treatment [115]. This data provided the rationale to combine cisplatin and guadecitabine in a phase Ib study of heavily pretreated cisplatin-resistant TGCT patients [NCT02429466]. This combination was tolerable and showed two major responses and an overall clinical benefit rate of 46% from 14 patients [127].

### 4.3. Histone Modifications

Compared to DNA methylation, relatively less is known concerning histone modifications in TGCTs. There is some data to suggest that like pluripotent embryonic stem and induced pluripotent stem cells, TGCTs and especially EC, may have high levels of bivalent histone marks H3K27me3 and H3K4me3 [128,129]. Bivalent marks are proposed to define genes that are poised to be either activated or permanently silenced upon differentiation [128,129]. This predicts that TGCT subtypes that are more differentiated will have a decrease in bivalency. While studies characterizing the actual genome-wide distribution of histone marks in TGCTs are lacking, there have been a number of studies assessing the expression of various histone writers, erasers and readers and related studies suggesting these expression levels may be exploited therapeutically [30,31,32,120,130]. In general, and as with DNA methylation inhibitors, there is evidence that TGCT cells may be especially sensitive to histone targeting drugs due to their pluripotent nature and that the resultant anticancer effects are multifactorial and include an apoptosis/stress response, alterations in gene expression and differentiation, and loss of pluripotency [30,31,32,120,130]. 

The HDAC inhibitors belinostat and panobinosat among others have antitumor effects in both cisplatin sensitive and resistant EC cell lines and xenografts [131,132]. In vitro and in vivo antitumor effects in cisplatin resistant TGCT cell lines were also demonstrated for the dual HDAC and cytoskeletal inhibitor, animacroxam [133]. Interestingly, the HDAC inhibitor romidepsin was shown to cause toxicity at low doses in cisplatin resistant and sensitive EC and seminoma cells but not in fibroblasts or Sertoli cells and effects were associated with the regulation of the SWI/SNF-complex member, ARIDIA [134,135]. The seminoma cell line Tcam-2 was sensitive to the HDAC inhibitor depsipeptide [136].

Apart from HDAC inhibitors, EC and seminoma cells are also sensitive to the bromodomain inhibitor JQ1 and inhibitors of LSD1, a H3K4 demethylase [137,138]. Interestingly, TGCTs, especially seminoma, overexpress LSD1 [137]. We showed by RNA-seq and gene set enrichment analysis that a panel of isogenic cisplatin resistant EC lines have a dramatic enrichment in genes normally repressed by H3K27me3 and the polycomb repressive complex which correlated with a substantial decrease in global H3K27me3 and decreased expression of EZH2 and BMI1 [139]. Importantly, repression of H3K27 methylation with the EZH2 inhibitor GSK126 conferred cisplatin resistance to parental cells while induction of H3K27 methylation with the histone lysine demethylase inhibitor GSKJ4 resulted in increased cisplatin sensitivity to resistant cells, suggesting that the polycomb pathway is involved in the regulation of cisplatin sensitivity of TGCT cells [139]. As stated above, follow up analysis of these cisplatin resistant models suggest the same polycomb target genes are coordinately regulated by both H3K27 methylation and DNA methylation [113]. In summary, studies on epigenetic targeting of TGCTs suggest that tumorigenicity in this pluripotent, germ cell context may be especially vulnerable to epigenetic alterations compared to somatic cancers.

### 4.4. Non-Coding RNA

A third major epigenetic pathway, non-coding RNA, is a largely unexplored area in TGCT research but has the potential to provide insight on TGCT biology and to provide potential therapeutic targets. Most of the progress so far is in the area of biomarkers since it is clear that TGCTs overexpress unique non-coding RNAs, for reviews see [140,141]. For example, a highly promising miRNA, miR371a-3p is poised to enter the clinic alone or in combination with other miRNAs as a plasma biomarker of TGCT burden since strong evidence suggests superior sensitivity and specificity compared to standard-of-care serum biomarkers AFP and hCG [140,141]. Similar to chromosome 12p, miR371a-3p appears pathognomonic for TGCTs, but also like 12p (first discovered in the 1980s) its functional role is very poorly understood. Studies have also explored whether miRNAs could be used to detect GCNIS or discriminate between malignant TGCT and benign teratoma [141,142,143]. However, despite this progress, the functional role of these TGCT-specific miRNAs is very poorly understood, although a few pioneering studies on a handful of miRNAs have explored functional roles including the finding that miR-372 and miR-373 neutralized p53 function through inhibition of the tumor suppressor LATS2 in TGCT cells [144,145,146,147]. Another form of non-coding RNA is the PIWI pathway that has a physiologic role in male germ cells. There is evidence that the PIWI pathway is altered/abrogated in TGCTs, although more work will be required to precisely define these alterations and their functional roles [99,100].

In summary, the epigenetic states of TGCTs and their association with key aspect of their biology, tumorigenicity, pluripotency, and sensitivity and resistance to chemotherapeutics are really just beginning to be appreciated. A complete and more precise understanding will be required to fully utilize epigenetics as tools and therapeutic targets in TGCT clinical medicine.

## 5. Rock and a Hard Place Model

**Figure 2:**
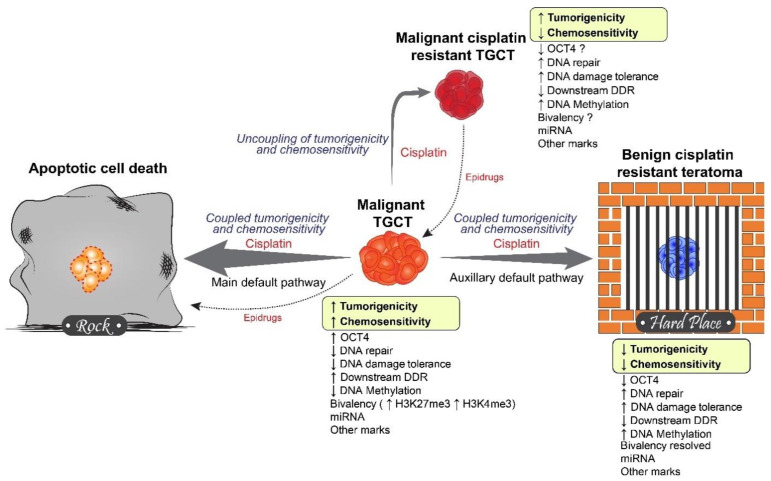
Rock and a hard place model of TGCT chemosensitivity. The model speculates that an explanation for why TGCTs are so sensitive to cisplatin-based therapy compared to other solid tumors is that the very epigenetic state(s) that drives tumorigenicity are the same or “linked” to the epigenetic state(s) that drive chemosensitivity. In such a model there are two main outcomes for cisplatin treated TGCTs; to either die or differentiate (in the extreme case to benign teratoma). This situation is predicted not to occur in other solid tumor types, or their cancer stem cells. In rare cases the epigenetic state driving tumorigenicity of TGCTs becomes uncoupled from the epigenetic state driving chemosensitivity resulting in cisplatin resistant TGCTs that retain tumorigenicity. Epigenetic drugs have the potential to “recouple” chemosensitivity with tumorigenicity or to target unique epigenetic vulnerabilities of TGCTs directly. Epigenetic drugs that cause teratoma formation would not be optimal as teratoma is still a clinical issue necessitating surgical removal.

Although there is still much to learn about the potentially unique role of epigenetics in TGCTs we have developed a “rock and a hard place” or “differentiation or die” model of TGCT epigenetic states that could be consistent with both the suspected origins of TGCTs and their unique curability (Figure 2). This model predicts that TGCTs are unique compared to other solid tumors in that the very epigenetic state that drives tumorigenicity of TGCTs is the same epigenetic state that drives chemosensitivity to cisplatin. In other words, the epigenetic state driving tumorigenesis of TGCTs is coupled to the epigenetic state driving chemosensitivity. This epigenetic state is likely complex and involves both distinct global DNA methylation status, H3K27me3, and H3K4me3 status (bivalency status) and other epigenetic mechanisms like miRNA, histone acetylation, and chromatin remodeling complexes. These epigenetic mechanisms are likely also intimately interconnected and involve reciprocal regulations of shared genomic regions.

The model predicts that there are two main outcomes or “default pathways” for cisplatin treated TGCTs. The most common pathway is apoptotic death due to the increased chemosensitivity directly attributed to the epigenetic state. The second default pathway is for the epigenetic state to adjust under selective pressure to afford resistance to cisplatin. However, since for TGCTs the epigenetic state of tumorigenicity is linked to the epigenetic state of chemosensitivity, this results in simultaneous loss of tumorigenicity. This can take the form, for example, of the differentiation seen in a subset of patients that present with benign cisplatin resistant teratoma after cisplatin therapy (Figure 2). In a small subset of TGCT patients a rare event occurs. Similar to the baseline situation for most other solid tumors, the factors driving tumorigenesis of TGCTs have now become uncoupled from the epigenetics driving chemosensitivity. In this case TGCT tumorigenicity is no longer comprised in the setting of cisplatin resistant. In this scenario seminoma that is generally less prone to cisplatin resistance compared to nonseminoma would be less prone to uncoupling.

What constitutes the coupling mechanism? There are several possibilities. There are many recent findings suggesting that the epigenetic chromatin context can directly influence the efficiency of DNA repair, the DNA damage response and DNA damage tolerance of cells [148,149,150]. Hence, the very epigenetic context that affords pluripotency (linked to tumorigenicity in TGCTs) also is the epigenetic state most sensitive to DNA damage. Other more direct mechanisms involve epigenetic regulation of components of the DNA repair and DNA damage response pathways. For example, DNA promoter methylation of *BRCA1*, *RAD51*, *MLH1*, and *MGMT* has been shown to occur in and to influence chemosensitivity and progression in TGCTs [95,97,116]. 

Anecdotally we have observed during development of cisplatin resistant clones of TGCT derived EC cells that some cisplatin resistant clones have a change in morphology (in other words partially differentiated). However, a number of cisplatin resistant cells retain normal morphology. We have found that some of these later clones have a partial decrease in tumorigenicity while others maintain parental tumorigenicity levels (Spinella, manuscript in preparation). This implies that in certain situations chemosensitivity can be uncoupled from tumorigenicity (or pluripotency) in TGCT cells. It is unclear whether the uncoupling mechanism would be driven by epigenetic or genetic alterations or a combination of both. Finally, the model predicts that the unique epigenetic state of TGCTs affords unique vulnerabilities to epigenetic drugs that could mediate recouping or direct apoptotic cell death (Figure 2).

## 6. Conclusions

In this review we have tried to make the case that epigenetics are exceptionally key drivers of TGCT biology compared to other more common somatic cancers. The reality is that mechanisms of TGCT initiation, progression, and response to therapy are likely multifactorial and involve an interaction between genetic and epigenetic factors. Still, what we know about TGCT thus far, especially aspect of their epidemiology, origins, low mutational burden, and lack of targeted therapeutics does suggest that these tumors may have a unique dependence on distinct epigenetic programs. Unique dependence may also provide unique vulnerabilities to targeted intervention of epigenetic pathways.

## Data Availability

Not applicable.
